# Supplementary material for: Lactobacillus salivarius CML352 Isolated from Chinese Local Breed Chicken Modulates the Gut Microbiota and Improves Intestinal Health and Egg Quality in Late-Phase Laying Hens
Source: Microorganisms. 2022 Mar 28;10(4):726. doi: 10.3390/microorganisms10040726 (PMC9029475; doi:10.3390/microorganisms10040726)
Supplement: Supplementary file 1 [file microorganisms-10-00726-s001.zip › microorganisms-1642304-supplementary.pdf]

Table S1. Basic information of two *Lactobacillus salivarius* strains in RAST server.

	JSWX5_1	CML352
Cofactors, Vitamins, Prosthetic Groups, Pigments	48	46
Cell Wal and Capsule	47	46
Virulence, Disease and Denfense	37	33
Potassium metabolism	3	3
Photosynthesis	0	0
Miscellaneous	10	8
Phages, Prophages, Transposable elements, Plasmids	0	3
Membrane Transport	19	19
Iron acquistion and metabolism	4	4
RNA Metabolism	33	33
Nucleosides and Nucleotides	73	84
Protein Metabolism	114	111
Cell Division and Cell Cycle	5	5
Motility and Chemotaxis	0	0
Regulation and Cell signaling	8	13
Secondary Metabolism	0	0
DNA Metabolism	50	68
Fatty Acids, Lipids, and Isoprenoids	22	22
Nitrogen Metabolism	0	0
Dormancy and Sporulation	6	6
Respiration	17	17
Stress Response	9	10
Metabolism of Aromatic Compounds	4	3
Amino Acids and Derivative	108	97
Sulfur Metabolism	3	3
Phosphorus Metabolism	9	9
Carbohydrates	146	121

Table S2. Predicted antimicrobial peptides produced by *L. salivarius* CML352 and the target pathogens.

**Target pathogen**	Antimicrobial peptide **ID**	**Class**	**Predictive value (Type)**
E. coli	BDGMGDOC_00461	Active	0.53 (PPV)
	BDGMGDOC_00473	Active	0.53 (PPV)
	BDGMGDOC_00600	Active	0.50 (PPV)
	BDGMGDOC_00650	Active	0.56 (PPV)
	BDGMGDOC_00892	Active	0.58 (PPV)
	BDGMGDOC_01260	Active	0.52 (PPV)
	BDGMGDOC_01293	Active	0.61 (PPV)
	BDGMGDOC_01453	Active	0.51 (PPV)
	BDGMGDOC_01709	Active	0.50 (PPV)

S. typhimurium	BDGMGDOC_00144	Active	0.588 (PPV)
	BDGMGDOC_00180	Active	0.897 (PPV)
	BDGMGDOC_00473	Active	0.578 (PPV)
	BDGMGDOC_00650	Active	0.51 (PPV)
	BDGMGDOC_00892	Active	0.998 (PPV)
	BDGMGDOC_00892	Active	0.998 (PPV)
	BDGMGDOC_01293	Active	0.97 (PPV)
	BDGMGDOC_01517	Active	0.65 (PPV)
	BDGMGDOC_01910	Active	0.68 (PPV)
C. perfringens	BDGMGDOC_00892	Active	0.534 (PPV)
	BDGMGDOC_01910	Active	0.564 (PPV)
